# Cerebral oxygenation in 45-degree trendelenburg position for robot-assisted radical prostatectomy: a single-center, open, controlled pilot study

**DOI:** 10.1186/s12894-020-00774-4

**Published:** 2020-12-30

**Authors:** Clemens Wiesinger, Dominik Stefan Schoeb, Mathias Stockhammer, Emir Mirtezani, Lukas Mitterschiffthaler, Helga Wagner, Johann Knotzer, Walter Pauer

**Affiliations:** 1 Department of Urology, Wels-Grieskirchen Medical-Center, Grieskirchner Straße 42, 4600 Wels, Austria; 2 Department of Urology, Faculty of Medicine, Medical Center – University of Freiburg, Hugstetterstr. 55, 79106 Freiburg, Germany; 3 Department of Urology, BHB Salzburg, Kajetanerplatz 1, 5010 Salzburg, Salzburg Austria; 4 Department of Anesthesiology and Intensive Care II, Wels-Grieskirchen Medical Center, Grieskirchner Straße 42, 4600 Wels, Austria; 5 Medical Statistics and Biometry, Institute for Applied Statistics, Johannes Kepler University Linz, Altenbergerstrasse 69, 4040 Linz, Austria

**Keywords:** Robotic surgical procedures/adverse effects, Head-down tilt, Robotic surgical procedures/methods, Prostatectomy/adverse effects*, Laparoscopy/adverse effects, Monitoring, Intraoperative

## Abstract

**Background:**

Within the last decade, robotically-assisted laparoscopic prostatectomy (RALP) has become the standard for treating localized prostate cancer, causing a revival of the 45° Trendelenburg position. In this pilot study we investigated effects of Trendelenburg position on hemodynamics and cerebral oxygenation in patients undergoing RALP.

**Methods:**

We enrolled 58 patients undergoing RALP and 22 patients undergoing robot-assisted partial nephrectomy (RAPN) (control group) in our study. Demographic patient data and intraoperative parameters including cerebral oxygenation and cerebral hemodynamics were recorded for all patients. Cerebral function was also assessed pre- and postoperatively via the Mini Mental Status (MMS) exam. Changes in parameters during surgery were modelled by a mixed effects model; changes in the MMS result were evaluated using the Wilcoxon signed rank test.

**Results:**

Preoperative assessment of patient characteristics, standard blood values and vital parameters revealed no difference between the two groups.

**Conclusions:**

Applying a 45° Trendelenburg position causes no difference in postoperative brain function, and does not alter cerebral oxygenation during a surgical procedure lasting up to 5 h. Further studies in larger patient cohorts will have to confirm these findings.

***Trial registration*:**

German Clinical Trial Registry; DRKS00005094; Registered 12th December 2013—Retrospectively registered; https://www.drks.de/drks_web/navigate.do?navigationId=trial.HTML&TRIAL_ID=DRKS00005094.

## Background

Since Prof. Friedrich Trendelenburg developed the “Raised Pelvic Position” for abdominal and pelvic operations in the 1880s, and its first description by his student Dr. Willy Meyer in 1885, the term “Trendelenburg Position” has become the standard nomenclature to describe a patient lying in supine position [1]. While originally intended to facilitate surgical procedures when treating vesicovaginal fistulae [2] and later for other gynecological operations, the patient was placed on a table tilted 45° downwards with legs and feet over the table’s edge [1]. This “extreme” form of the Trendelenburg position, however, was later abandoned mostly, and while the “Raised Pelvic Position” served various purposes such as treating venous air embolism [3] or facilitating intestinal surgery in obese patients [4], the angle applied usually did not exceed 30°. Furthermore, while the Trendelenburg position was traditionally employed for the acute treatment of hypotension, clinical studies had not yielded any evidence of this method’s beneficial effect on the patient [5]. The original Trendelenburg Position using a 45° angle has recently experienced a revival in the operation theater, with robot-assisted procedures becoming the new standard in minimally invasive surgery. After its first description in 2000 [6, 7], robotically-assisted laparoscopic radical prostatectomy has become the new standard surgical therapy for localized prostate cancer [8] and since then, robotically-assisted surgery has been extended to various other procedures such as hysterectomy [9], proctocolectomy [10], nephrectomy [11], and more.

To perform robot-assisted laparoscopic prostatectomy, an “extreme” 45° Trendelenburg position is often applied, continuing throughout the procedure. In addition to abdominal CO2-insufflation, significantly reduced pulmonary compliance and upper airway edema have been reported [12].

In this study we explore the influence of a prolonged Trendelenburg Position during robotically-assisted radical prostatectomy on intraoperative hemodynamics and cerebral oxygenation, as well as pre- and postoperative cerebral function.

## Methods

### Patient cohort and study protocol

We enrolled in our study a total of 58 consecutive male patients (study group) who underwent robot-assisted radical prostatectomy (RALP) for localized prostate cancer therapy and 22 consecutive male patients undergoing robot-assisted partial nephrectomy (RAPN) (control group) for suspected kidney-cell carcinoma at the Wels-Grieskirchen Medical Center. We chose the RAPN procedure for our control group since it is a routine, robotically-assisted procedure at our institution, and a horizontal position is maintained during the entire procedure. Preoperative oncological staging and the indication for the above-mentioned surgical intervention were determined according to the current guidelines of the European Association of Urology (EAU) on prostate cancer and renal cell carcinoma. General anesthesia was induced with 150–200 µg of fentanyl and 150–200 mg of propofol then 0.6 mg/kg of rocuronium was administered followed by orotracheal intubation. General anesthesia was maintained with sevoflurane (MAC 0.9–1.2) and continuous infusion of remifentanil (1–2.5 µg/kg/min). Pneumoperitoneum was induced by insufflating CO2 with gas pressure set to 8 mmHg during the whole operation. After pneumoperitoneum was achieved, study patients were positioned in 45° Trendelenburg by tilting the operating table. Control group patients were brought into flank position prior to inducing pneumoperitoneum with the operation table slightly flexed. Once the procedure started, this patient position was maintained during the entire procedure. All surgeries were performed by experienced urologists (> 100 robot-assisted procedures) using the da Vinci® Si Surgical System (Intuitive Surgical, Sunnyvale, CA, USA) with the surgeon performing the procedure at a control table located away from the operating table. Patients provided informed consent prior to inclusion in our study, which was approved by our local ethics committee (ethics committee of Upper Austria (Study Nr. O-18-13)); it was therefore conducted in accordance with the ethical standards laid down in the 1964 Declaration of Helsinki and its later amendments. Our sole exclusion criterion was involvement in any other clinical study, but this did not apply since no patients screened met this criterion. The study was designed and conducted in accordance with the consort criteria.

### Intraoperative monitoring and data collection

Patient demographics and ASA classification was recorded 24–6 h prior to surgery. In addition, mini-mental status test (MMS) was carried out, and a standard blood workup (hemoglobin, hematocrit, blood coagulation parameters) was obtained. Furthermore, we documented baseline blood pressure and heart frequency. Intraoperative heart frequency (HF), cerebral oxygenation (rSO2), and blood pressure (RR) were obtained and recorded prior to final positioning and consecutively at 10-min intervals throughout the procedure. Also the FiO2 was (= fraction of inspired oxygen) was monitored. To measure rSO2, bilateral cerebral oximetry sensors were placed on the patient’s forehead and rSO2 was continuously monitored using near infrared spectroscopy (NIRS) and the INVOS analytics tool for recording. We also recorded total operating time, time in Trendelenburg position and time of robotic actuation. Within 6–24 h after surgery, standard blood panel was repeated and all patients underwent a MMS Test. Complications were classified according to the Clavien Dindo classification and recorded over a 3 months follow up for all included patients.

### Statistical evaluation

This trial was designed to explore whether a 45 Degree Trendelenburg Position causes any differences in intraoperative clinical parameters, or changes in the MMS exam result in comparison with patients not in a head-down position. Patients were included in this exploratory study applying the intention-to-treat principle. All statistical calculations were performed by an experienced and licensed statistician (H.W.). Changes in the MMS exam outcome before and after surgery were subjected to the Wilcoxon rank-sum test. To analyze the evolvement of parameters during surgery, we made an exploratory comparison of the different variables at the same time point, followed by a longitudinal analysis of a variable’s evolvement, and investigated the change in a given variable compared to its initial value by linear mixed effects models. To analyze changes, the response variable’s pre-operation value centered at a reasonable value and that variable’s interaction with time were included in the model. All statistical computations were performed using the R statistics software (Version 3.5.2). All tests were two-sided with p < 0.05 considered to indicate statistical significance, but as this is not a confirmatory trial, significant results can be interpreted only as indicating a potential association.

## Results

### Patient characteristics and preoperative results

All patients included in our study underwent a complete evaluation, and we observed no drop outs. Median age in our study group was 66, ranging from 48 to 79 years. Our control group’s median age was 56 years (45–84). ASA classification showed a median of 2 for both groups. Routine blood panel revealed a median hemoglobin value of 15.3 g/dl (13–17.1) for the RALP group and 14 g/dl (10.20–16.30) for the RAPN group. Median preoperative RR was 137.5 (94–210)/80 (60–110) for our study group and 146.5 (100–180)/80 (60–103) for the control group. The median HF measured 66 bpm (45–92) (RALP) and 70 bpm (60–100) (RAPN). No significant difference was detected in individual preoperative patient characteristics (Table 1) except for the RALP group’s higher hemoglobin value.

**Table 1:** Patient demographics and baseline parameters recorded preoperatively

	RALP	RAPN	p value
Patients total	58	22	–
BMI (kg/m^2^)	27.5 (16.41–41.36)	26.7 (21.56–33.95)	0.75
Weight (kg)		79 (48.5–110)	0.57
Height (cm)		171.5 (150–185)	0.45
Hemoglobin (g/dl)		14.00 (10.20–16.30)	**0.0008**
RRsyst (mmHg)		146.5 (100–180)	0.21
RRdiast (mmHg)		80 (60–103)	0.85
HF(bpm)		70 (60–100)	0.08

All data is given as median with range (where applicable), significant p-values are given in bold

*RALP* robot assisted radical prostatectomy, *RAPN* robot assisted radical nephrectomy

The preoperative MMS exam revealed a median point value of 30 (IQR = 1) for RALP and a point value of 30 (IQR = 0.75) for RAPN. There was no statistically significant difference here, either (p = 0.29).

### Perioperative results and correlation with patient characteristics

**Fig. 1:**
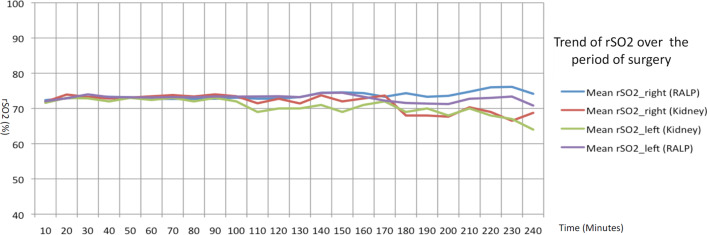
Mean oxygen saturation over the course of surgery for all performed operations

Total median operation time was 180 min (IQR = 56.75; documentation for 2 procedures missing) for the RALP group and 140.5 min (IQR = 76.50) for the RAPN group, thus the study group’s surgery time was longer (p = 0.01). Also, the RALP group’s robotic-actuation time (= console time) was significantly longer than the RAPN group’s (p = 0.001). RR values exhibited no difference in systolic values, but the prostate group’s diastolic values were significantly higher, although that effect vanished after about 120 min’ operation time. However, since the RAPN group’s operation time was shorter, we had access to fewer measurements after 120 min, with only 1 measurement at 210 min. Our analysis of any statistical change in systolic RR compared to the initial value showed no group difference either. We noted a significant difference between the RALP and RAPN group in diastolic RR compared to the initial valued (p = 0.00), with the prostate group’s RR values significantly higher 120 min into the operation. Age also seemed to reveal an effect on the change in diastolic RR, with the patients over 60 showing significantly lower values (p = 0.00). HF during surgery did not differ significantly between groups in either the longitudinal analysis or analysis of change during the operation. We did, however, observe a tendency for older patients (> 60 years) to present a lower heart frequency. Intraoperative FiO2 measurements revealed very little variation, with no group difference in the intraoperative change versus the initial value. Although the former was also not associated with patient BMI, higher-BMI patients revealed a generally higher FiO2 (p = 0.01). The pCO2 (mean overall change: − 0.73 ± 0.18; p < 0.001) value and pO2 (mean overall change: 0.95 ± 0.09; p < 0.001) and rSO2 value (mean change: − 0.18 ± 0.08; p < 0.03) showed a significantly smaller change in patients with an initially higher value (see also Fig. 1). Patient characteristics demonstrated no significant influence in these values or any difference between study groups.

### Postoperative examination and follow up results

Patients were monitored for perioperative and postoperative complications until 3 months after surgery. A total of 22 complications were recorded within the RAPN group and classified according to the Clavien Dindo classification: 10 were classified as category 1, 4 as category 2, 3 as 3a, and 4 as 3b. There were no category 4 or 5 complications. All complications were closely analyzed to determine whether any had been specifically caused by or related to patient positioning, but none were detected.

**Fig. 2:**
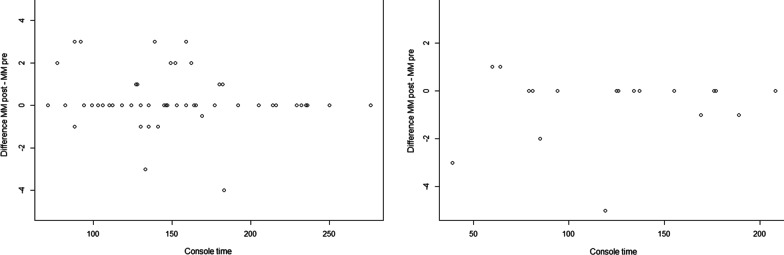
Difference in point value of pre- and postoperatively Mini Mental Status exam for both groups

Postoperative repetition of the MMS exam yielded no significant difference compared to the preoperative value in the RALP group (p = 0.16) and RAPN group (p = 0.19), suggesting no negative effect of the operation and intraoperative positioning on cognitive function (Fig. 2).

## Discussion

In the current study we compared intraoperatively recorded hemodynamics of patients undergoing Robot-Assisted Radical Prostatectomy and Robot-Assisted Partial Nephrectomy. We documented no relevant preoperative difference in patient characteristics. Analysis of these data, as well as the comparison of both groups showed decreasing blood pressure and slightly elevated pCO2, as well as a slight decrease in pO2 in both groups, as would be expected during a laparoscopic surgical intervention. However, besides slightly higher diastolic blood pressure, hemodynamic monitoring showed no significant difference within the two groups, which suggests that the Trendelenburg position exerted a negligible effect in the RALP group on patients’ hemodynamics. In addition, noninvasive cerebral oxygenation monitoring revealed no significant change during the operation, and no difference between our two groups. This finding is supported by other studies applying infrared spectroscopy. In a study by Park et al., intraoperative measurements revealed a statistically significant increase in rSO2 when keeping the pCO2 constant [13]. Other studies report unchanged rSO2, as our data demonstrate [14].Multiple physiological reactions should be considered in this context. On one hand, there is evidence that intracranial pressure rises during Trendelenburg position due to increased venous pressure [15]. This effect is complemented by the increase in intraabdominal pressure due to the pneumoperitoneum, which also increases intracranial pressure by obstructing the venous return [16]. However, pneumoperitoneum also increases cerebral perfusion by raising pCO2 and releasing catecholamine, causing improved cerebral blood flow and in turn potentially higher rSO2 [17]. The net effect of this physiological reactions, as described by Tanaka et al., is a slight increase in cerebral blood volume [18] that was, however, reported to be less than 10%. We found no changes in rSO2 in this study. Our study data indicate that the Trendelenburg position exerts an only slight effect on rSO2, and no signs of impaired cerebral oxygenation.

Follow up of all patients including Mini Mental Status evaluation of the cognitive state also showed no negative effect on the intellectual ability of all operated patients in both groups. In addition, we detected no complications related to the Trendelenburg positioning. We therefore conclude that the Trendelenburg position as applied in Robot-Assisted Radical Prostatectomy is safe for the patient and can be applied during surgical interventions requiring extended operating times. Our study however, has some limitations. First of all, it was exploratory and conducted only on a small cohort; further prospective studies will have to confirm these results. In addition, our control group patients did not undergo the same surgical intervention, since Robot-Assisted Radical Prostatectomy is currently only performed in Trendelenburg position, thus a control group undergoing the same procedure was not possible. Furthermore, our control group included, besides partial nephrectomy, various other Robot-Assisted procedures on the kidney including one radical nephrectomy, one adrenalectomy and a pyeloplasty. As the adrenalectomy was due to a benign hormone-inactive tumor, we cannot rule out that surgical adrenal manipulation might have altered our results.

## Conclusions

The 45° Trendelenburg position demonstrated no negative effect on postoperative brain function and did not alter cerebral oxygenation during a surgical procedure lasting up to 5 h in this exploratory study. Further studies in larger patient cohorts will have to confirm these findings.

## Data Availability

The full dataset generated and analyzed during the current study are available from the corresponding author on reasonable request.

## Abbreviations

ASA: American Society of Anesthesiologists
BMI: Body Mass Index
CA: California
FiO2: Fraction of inspired oxygen
HF: Heart frequency
IQR: Interquartile range
MMS: Mini-mental status
NIRS: Near infrared spectroscopy
RALP: Robotically-assisted laparoscopic prostatectomy
RAPN: Robot-assisted partial nephrectomy
RR: Blood pressure
rSO2: Cerebral oxygenation
